# Eunkyosan for treatment of the common cold: A protocol for the systematic review of controlled trials

**DOI:** 10.1097/MD.0000000000010527

**Published:** 2018-05-04

**Authors:** Hesol Lee, Bohyung Kang, Jun-Yong Choi, Sunju Park, Myeong Soo Lee, Ju Ah Lee

**Affiliations:** a Department of Internal Medicine College of Korean Medicine, Gachon University, Seongnam, Korea; b Korean Medicine Hospital of Pusan National University, Yangsan; c Department of Preventive Medicine, College of Korean Medicine, Daejeon University; d ???.

**Keywords:** common cold, Eunkyosan protocol, systematic review

## Abstract

**Introduction:**

Eunkyosan (EKS) is widely used for common colds in East Asian countries. Many clinical trials assessing the efficacy and safety of EKS formula for the treatment of common colds have been reported. This review will assess the clinical evidence for and against the use of EKS formula as a treatment for common colds.

**Methods and analysis:**

Fourteen databases will be searched from inception until March 2018. We will include randomized controlled trials (RCTs) assessing EKS decoctions for any type of common cold. All RCTs of decoctions or modified decoctions will be included. The methodological qualities of the RCTs will be assessed using the Cochrane Collaboration tool for assessing risk of bias, while confidence in the cumulative evidence will be evaluated using the Grading of Recommendations Assessment, Development and Evaluation (GRADE) instrument.

**Ethics and dissemination:**

This systematic review will be published in a peer-reviewed journal and will also be disseminated electronically and in print. The review will be updated to inform and guide healthcare practices.

**Registration number:** CRD42018087694

## Abstract 2

Supplemental Digital Content is available in the text

## 1 Introduction

The common cold is an infectious viral disease of the upper respiratory tract that primarily affects the nose.^[1]^ The common cold, a conventional term for a mild upper respiratory tract infection (URTI), is a common and frequent respiratory disease mainly caused by a virus.^[2,3]^ Cold symptoms are related to the infected mucosa, typically peaking at 1 to 3 days and lasting 7 to 10 days, although they can persist for 3 weeks.^[4–7]^ They include sore throat, rhinitis, rhinorrhea, cough, and malaise.^[5,7]^

Although the common cold is usually mild and self-limiting, with a mean duration of 7 to 10 days,^[8,9]^ its economic burden on society is significant in terms of visits to health care providers, treatments, and absences from work and school.^[10,11]^ In addition, in some cases, viral pathogens may spread to adjacent organs, resulting in different clinical manifestations, and occasionally, colds cause bacterial complications.^[5]^ Its impact on society and health care is large. Of individuals with an upper respiratory tract infection, 7% to 17% of adults^[10,12]^ and 33% of children^[10]^ visit a physician. Upper respiratory tract infections result in an estimated increase of 12.5% in patient visits per month during cold and flu season.^[13]^

Antibiotics commonly used in respiratory infections have adverse effects such as nausea, vomiting and diarrhoea, and these toxins are known to exhibit dose dependency.^[14]^ Antibiotics are prescribed at > 100 million adult ambulatory care visits annually.^[15]^ Inappropriate antibiotic use or overuse for common cold is an important contributor to antibiotic resistance, an urgent public health threat.^[16]^ Antibiotic use is the primary driver of antibiotic resistance^[17]^ and leads to adverse events ranging from allergic reactions to *Clostridium difficile* infections.^[18]^ In places with a greater prescribing of broad-spectrum antibiotics, rates of multidrug-resistant pneumococcal disease are higher.^[19]^

In places with greater prescribing of broad-spectrum antibiotics, specifically extended-spectrum cephalosporins and macrolides, rates of multidrug-resistant pneumococcal disease are higher.^[3]^ In places with greater prescribing of broad-spectrum antibiotics, specifically extended-spectrum cephalosporins and macrolides, rates of multidrug-resistant pneumococcal disease are higher. In places with greater prescribing of broad-spectrum antibiotics, specifically extended-spectrum cephalosporins and macrolides, rates of multidrug-resistant pneumococcal disease are higher.^[3]^ Antibiotic-resistant infections affect 2 million people and are associated with 23,000 deaths annually in the United States, according to the Centers for Disease Control and Prevention (CDC).^[20]^ Antibiotics are prescribed at >100 million adult ambulatory care visits annually, and 41% of these prescriptions are for respiratory conditions.^[1]^ Inappropriate antibiotic use for ARTI is an important contributor to antibiotic resistance, an urgent public health threat. Thus, it is urgent to develop a method that can prevent the overuse or abuse of antibiotics and increase the efficacy of antibiotics. Eunkyosan (EKS) is widely used in traditional Korean medicine (TKM) and traditional Chinese medicine (TCM) for treating the symptoms of respiratory diseases in oriental medicine.^[21]^ In this review, we will systematically review randomized controlled trials (RCTs) to assess the effectiveness and safety of EKS for the treatment of common colds.

## 2 Methods

### 2.1 Study registration

This study will follow the guidelines outlined in the Preferred Reporting Items for Systematic Reviews and Meta-Analysis (PRISMA) statement for meta-analyses of healthcare interventions^[22]^; additionally, the current protocol report adheres to the PRISMA Protocols (PRISMA-P).^[23]^ The protocol for this systematic review has been registered on PROSPERO 2018, under the number CRD42018087694.

### 2.2 Ethical approval

Because this study is not a clinical study, ethical approval is not required.

### 2.3 Data sources

The following databases will be searched from inception to the present date: MEDLINE, EMBASE, the Cochrane Central Register of Controlled Trials (CENTRAL), AMED, and CINAHL. We will also search 6 Korean medical databases (OASIS, the Korean Traditional Knowledge Portal, the Korean Studies Information Service System, KoreaMed, the Korean Medical Database, and DBPIA) and 3 Chinese databases, including CNKI (the China Academic Journal, the China Doctoral Dissertations and Master's Theses Full-text Database, the China Proceedings of Conference Full-Text Database, and the Century Journal Project), Wanfang, and VIP. In addition, we will search a Japanese database and conduct nonelectronic searches of conference proceedings. The search strategy that will be applied in the MEDLINE database is presented in Supplement 1;. Similar search strategies will be used in the other databases.

## 3 Types of studies

Prospective randomized controlled trials (RCTs) that evaluate the effectiveness of EKS for common colds will be included in this review. Both treatment with EKS alone and concurrent treatment with EGS and another therapy will be considered acceptable if EKS is applied to the intervention group only and any other treatment is provided equally to both groups. Trials with any type of control intervention will be included. No language restrictions will be imposed. Hard copies of all articles will be obtained and read in full.

## 4 Types of participants

All strains of common colds will be eligible for inclusion. Participants who have both common colds and accompanying diseases will be excluded. There will be no restrictions based on other conditions such as age, sex, or symptom severity.

## 5 Types of interventions

Studies that evaluate any type of formulation (i.e., decoction, tablet, pill, or powder) of EKS will be eligible for inclusion. The compositions of interventions will be reviewed, and interventions involving herbal combinations that differ from original EKS from the perspective of traditional East Asian medicine will be excluded from this review.

## 6 Data extraction and quality assessment

Hard copies of all articles will be obtained and read in full. Two authors (HL and BK) will perform the data extraction and quality assessment using a predefined data extraction form. In addition, all interventions applying acupuncture will be extracted using the Standards for Reporting Interventions in Clinical Trials of Acupuncture (STRICTA). Risk of bias will be assessed using the Cochrane Handbook risk of bias assessment tool version 5.1.0, which considers random sequence generation, allocation concealment, blinding of participants and personnel, blinding of outcome assessment, incomplete outcome data, selective reporting and other sources of bias.^[24]^ The results of the assessments will be presented using scores of “L,” “U,” and “H,” with “L” indicating a low risk of bias, “U” an uncertain risk of bias and “H” a high risk of bias. Disagreements will be resolved by discussion between all authors. When disagreements regarding selection cannot be resolved through discussion, an arbiter (JAL) will make the final decision.

## 7 Data collection and synthesis

### 7.1 Outcome measures

#### 7.1.1 Primary outcomes

Improved effectiveness including total treatment efficacy; that is, the number of patients whose common cold symptoms improve

#### 7.1.2 Secondary outcomes

Adverse events

Change in symptoms (e.g., sore throat, rhinitis, rhinorrhea, cough, and fever)

Information related to EKS usage

Pattern type in response based on TKM or TCM therapy

Range of dosage of EKS in each study

### 7.2 Assessment of bias in the included studies

We will independently assess the bias of the included studies according to the criteria in the Cochrane Handbook, version 5.1.0; these criteria include random sequence generation, allocation concealment, blinding of participants and personnel, blinding of outcome assessment, incomplete outcome data, selective reporting, and other sources of bias.^[24]^

### 7.3 Data synthesis

Differences between the intervention and control groups will be assessed. Mean differences (MDs) with 95% confidence intervals (CIs) will be used to measure the effects of treatment for continuous data. We will convert other forms of data into MDs. For outcome variables on different scales, we will use standard MDs (SMDs) with 95% CIs. For dichotomous data, we will present treatment effects as relative risks (RRs) with 95% CIs; other binary data will be converted into RR values.

All statistical analyses will be conducted using Cochrane Collaboration's software programme Review Manager (RevMan) version 5.3 (Copenhagen, The Nordic Cochrane Centre, the Cochrane Collaboration, 2014) for Windows. We will contact the corresponding authors of studies with missing information to acquire and verify the data whenever possible. When appropriate, we will pool the data across studies to conduct a meta-analysis using fixed or random effects. We will use GRADEpro software from Cochrane Systematic Reviews to create a Summary of Findings table.

### 7.4 Unit of analysis issues

For crossover trials, data from the first treatment period will be used. For trials that assessed more than one control group, the primary analysis will combine data from each control group. Subgroup analyses of the control groups will be performed. Each patient will be counted only once in the analyses.

### 7.5 Addressing missing data

Intention-to-treat analyses including all randomized patients will be performed. For patients with missing outcome data, the last observation carry-forward analysis will be performed. When individual patient data are initially unavailable, we will review the original source or the published trial reports for these data.

### 7.6 Assessment of heterogeneity

Based on the data analysis, we will use random- or fixed-effect models to conduct the meta-analysis. Chi-squared and *I*-squared tests will be used to evaluate the heterogeneity of the included studies. *I*^2^ values >50 will indicate high heterogeneity. When heterogeneity is observed, subgroup analyses will be conducted to explore the possible causes.^[1]^

### 7.7 Assessment of reporting biases

Funnel plots will be generated to detect reporting biases when a sufficient number of included studies (at least 10 trials) is available.^[25]^ However, as funnel plot asymmetries are not equivalent to publication biases, we will aim to determine the possible reasons for any asymmetries in the included studies, such as small-study effects, poor methodological quality, and true heterogeneity.^[25,26]^

## 8 Discussion

EKS is a widely used herbal medicine in various Asian countries, including China, Japan, and Korea. EKS consists of 9 herbal ingredients: *Forsythia suspensa* Vahl (40 g), *Lonicera japonica* Thunb. (40 g), *Arctium lappa* L. (24 g), *Mentha arvensis* L. var. *piperascens* Malinv. (24 g), *Platycodon grandiflorum* A. DC. or *Platycodon graucum* Nakai (24 g), *Phyllostachys bambusoides* Siebold & Zucc. (16 g), *Schizonepeta tenuifolia* Briq. or *Nepeta japonica* Maxim. (16 g), *Glycine max* Merrill (Leguminosae) (20 g), and *Glycyrrhiza uralensis* Fisch. or *Glycyrrhiza glabra* L. (20 g). EKS was first introduced in Wēn bìng tiáo biàn, a medical text written by Wu Ju Tong in China during the Qing Dynasty.^[21]^ In clinical practice, it is spicy and cold, has detoxifying properties when cooled and is used for respiratory system infections such as upper respiratory tract infection, bronchitis and various inflammatory diseases.^[21]^ EKS increased the survival rate and reduced lung viral titers in mice with an in vivo influenza virus infection model, which means that EKS has an active antiviral compound and an immunomodulatory compound.^[27]^ In China, there is a clinical report showing that a combination of maxingshigan-yinqiaosan (yinqiaosan; EKS) formula has a similar level of antipyretic effect as that of *oseltamivir* on the influenza A (H1N1) virus, a common infectious disease that was prevalent worldwide in 2009.^[28]^ There are various forms of EKS such as pills, capsules, tablets, and decoctions. All types of EKS will be included in this study. Currently, no systematic reviews of the effects of EKS formula on common colds have been published. This systematic review will provide a summary of the current evidence related to the effectiveness of EKS formula in the treatment of the symptoms of common colds. There are no systematic reviews that have been conducted for EKS for treating the common cold. Therefore, we will identify subtypes for which this remedy is particularly useful (e.g. certain pattern types based on TKM or TCM theory), as well as a range of dosages and modifications used to improve effectiveness, and will compare the duration of treatment in full review of this protocol. The original EKS formula was composed of the 9 herbs mentioned above; however, primary studies show high heterogeneity in ingredients of EKS. Therefore, we will investigate the composition of each formula used in the primary studies. This evidence will be useful to practitioners, patients and health policy makers regarding the use of acupuncture in common cold treatment.

## Author contributions

HL, JC, and JAL conceived the study, developed the criteria, searched the literature, analyzed the data, and wrote the protocol. JAL and MSL conducted the preliminary search. BK assisted in searching the Chinese literature and extracting the data. JAL and SP revised the manuscript. All authors have read and approved the final manuscript.

**Conceptualization:** Ju Ah Lee, Jun-Yong Choi.

**Funding acquisition:** Jun-Yong Choi.

**Methodology:** Ju Ah Lee.

**Writing – original draft:** Ju Ah Lee, Hesol Lee.

**Writing – review & editing:** Ju Ah Lee, Bohyung Kang, Sunju Park, Myeong Soo Lee.

## Supplementary Material

Supplemental Digital Content
