# Eunkyosan for treatment of the common cold: A protocol for the systematic review of controlled trials: Erratum

**DOI:** 10.1097/MD.0000000000012064

**Published:** 2018-08-17

In the article, “Eunkyosan for treatment of the common cold: A protocol for the systematic review of controlled trials”,^[1]^ which appeared in Volume 97, Issue 18 of *Medicine*, Dr. Myeong Soo Lee's affiliation should appear as Clinical Medicine Division, Korea Institute of Oriental Medicine, Daejeon, Republic of Korea.
