# Supplementary material for: Eunkyosan for treatment of the common cold: A protocol for the systematic review of controlled trials
Source: Medicine (Baltimore). 2018 May 4;97(18):e0527. doi: 10.1097/MD.0000000000010527 (PMC6392552; doi:10.1097/MD.0000000000010527)
Supplement: Supplemental Digital Content [file medi-97-e0527-s001.docx]

**Supplement 1. Search strategy for the MEDLINE database**

#1 Search "Common Cold"[Mesh]

#2 Search "common cold"[Title/Abstract]

#3 Search ('viral upper respiratory tract infection'[Title/Abstract] OR 'upper respiratory tract infection'[Title/Abstract])

#4 Search ("acute upper respiratory infection"[Title/Abstract]) OR "acute upper respiratory tract infection"[Title/Abstract]

#5 Search (coryza[Title/Abstract]) OR "coryza cold"[Title/Abstract]

#6 Search ('rhinovirus infection'[Title/Abstract] OR 'human rhinovirus'[Title/Abstract])

#7 Search 'paramyxovirus infection'[Title/Abstract]

#8 Search ('parainfluenza virus'[Title/Abstract] OR parainfluenza*[Title/Abstract])

#9 Search ('coronavirus'[Title/Abstract] OR 'coronavirus infection'[Title/Abstract])

#10 Search (#9) OR coronavir*[Title/Abstract]

#11 Search ('adenovirus'[Title/Abstract] OR 'human adenovirus infection'[Title/Abstract] OR adenovir*[Title/Abstract])

#12 Search ('respiratory syncytial pneumovirus'[Title/Abstract] OR 'respiratory syncytial virus infection'[Title/Abstract

#13 Search ('respiratory syncytial virus':ab,ti[Title/Abstract] OR 'respiratory syncytial viruses'[Title/Abstract])

#14 #1 OR #2 OR #3 OR #4 OR #5 OR #6 OR #7 OR #8 OR #10 OR #11 OR #12 OR #13

#15 Search "-"[Mesh]

#16 Search "-"[Title/Abstract]

#17 -[Title/Abstract]

#18 "Eunkyosan"[Title/Abstract]

#19 Search " 银翘散"[Title/Abstract]

#20 Search "Yinqiaosan"[Title/Abstract]

#21 Search (#15 OR #16 OR #17 OR #18 OR #19 OR #20)

#22 #14 AND #21
